# Additional findings in prostate MRI

**DOI:** 10.1186/s40644-025-00846-4

**Published:** 2025-03-11

**Authors:** Fabio Porões, Paraskevi Karampa, Thomas Sartoretti, Hugo Najberg, Johannes M. Froehlich, Carolin Reischauer, Harriet C. Thoeny

**Affiliations:** 1 University of Fribourg, Fribourg, Switzerland; 2 Department of Radiology, Cantonal Hospital of Fribourg, Chemin des Pensionnats 2-6 Case postale, Fribourg, CH-1708 Switzerland; 3 Department of Urology, Inselspital, University of Bern, Bern, Switzerland

**Keywords:** Magnetic resonance imaging, Incidental findings, Prostatic neoplasms, Neoplasm staging

## Abstract

**Background:**

Despite the increasing interest in abbreviated protocols, we adopted an extended protocol for all prostate MRIs. In this study, we assessed the benefits of an extended prostate MRI protocol, measured by the number and the clinical importance of additional findings (AFs) and their impact on patient management.

**Methods:**

In a single-center study, we retrospectively included 1282 patients undergoing prostate MRI between 01.10.2018 and 30.04.2022. Additional findings were defined as any pathology not located in the prostate or the seminal vesicles. These were classified as related or unrelated to prostate cancer (PCa). The latter were divided into groups based on low, moderate, or high clinical significance (group 1, 2, and 3). A finding unrelated to PCa was judged to be clinically significant (group 2: moderate, group 3: high) if further diagnostic investigations, or treatment was necessary. The degree of urgency of the latter determined moderate and high significance. For group 3 findings, a change in management was defined as further workup.

**Results:**

A total of 5206 AFs was recorded in 1240/1282 patients. One hundred and twenty-three (2.4% of all findings) extra-prostatic PCa related AFs were found in 106 (8.3% of all patients) patients. The remaining 5083 (97.6% of all findings) findings were not related to PCa, of which 3155 (60.6%), 1770 (34.0%), and 158 (3.0%) were assigned to groups 1, 2, and 3, respectively. A management shift was identified in 49 (3.8% of all patients) patients of group 3.

**Conclusion:**

The extended prostate MRI protocol shows a considerable prevalence of AFs of which more than a third are clinically significant, related or unrelated to PCa (groups 2 and 3). A substantial percentage (8.3%) of patients have extra-prostatic PCa-related AFs that change the patient’s disease stage and management. However, a change in management due to AFs unrelated to PCA that belong to group 3 is observed in less than 4% of all patients. The choice between extended and abbreviated prostate MRI protocols should be made based on available resources.

## Background

As part of the guidelines of the European Association of Urology, MRI prior to biopsy is of high relevance as it allows the detection of clinically significant prostate cancer (PCa) and locoregional staging [1]. Prostate MRI protocols may vary depending on the patients, the underlying clinical question, the management options, and the availability of MRI equipment. Institutions should optimise their imaging protocols based on equipment, capacity and expertise [2].

A distinction may be made between standard, abbreviated, and extended protocols. Standard protocols are performed according to Prostate Imaging Reporting and Data System (PI-RADS) recommendations [2]. They combine the anatomic information from T_1_ and T_2_-weighted sequences with functional information from diffusion-weighted imaging (DWI) and dynamic contrast-enhanced (DCE) imaging in the axial plane. They should also include at least one additional T_2_-weighted orthogonal plane (either in sagittal or coronal orientation) and one pulse sequence with a field of view (FOV) that permits evaluation of pelvic lymph nodes to the level of the aortic bifurcation [2, 3].

Abbreviated protocols have gained interest in recent years due to the limited MRI availability and the associated costs. Several approaches have been considered for protocol abbreviation [4]. The most common options are omitting DCE imaging, omitting acquisition of additional T_2_-weighted planes, and imaging of the prostate with only a restricted FOV. There is increasing evidence that these measures may allow for a substantial reduction in acquisition time without sacrificing diagnostic accuracy regarding detection of significant PCa [4].

Extended protocols include at least a cross-sectional abdominopelvic imaging sequence to assess for non-regional metastases [1]. In our institution, we adopted an extended protocol for all prostate MRIs with a coronal three-dimensional T_2_-weighted sequence of the abdomen and pelvis, which enables reconstructions in various planes. Such an extended protocol allows for the identification of additional findings (AFs) that would not be detected on the restricted FOV sequences of the abbreviated protocols. Even though most AFs are benign, some may explain patients’ symptoms or alter the primary treatment plan.

In the present work, we aim to retrospectively assess the frequency of AFs related and unrelated to PCa identified using an extended prostate MRI protocol, determine the clinical importance of AFs unrelated to PCa (low, moderate, or high), and their potential impact on the change in patient management. In addition, the distribution of moderately and highly clinically important AFs unrelated to PCa is correlated with the patients’ age and the PI-RADS score.

## Materials and methods

### Patient population

This retrospective, single-centre study was approved by the Cantonal Ethical Committee of “Canton de Vaud” (BASEC no. 2020 − 01859) with a waiver for written informed consent. All consecutive patients undergoing MRI of the prostate at our institution between 01.10.2018 and 30.04.2022 were included.

Indications for the MRI examination included: (a) rising and elevated prostate specific antigen (PSA) level (> 4 ng/mL), (b) suspicious digital rectal examination, (c) positive family history of PCa, (d) staging of a known PCa, (e) hematospermia, and (f) prostate infection.

Exclusion criteria included: (a) surveillance in patients with PCa who had undergone radical prostatectomy; (b) for patients on active surveillance, only the first MRI was included; (c) incomplete MRI protocol, for example due to claustrophobia or discomfort.

Patients’ age and PI-RADS score of the MRI examination were recorded.

### MRI protocol

Prostate MRI examinations were performed on a 3 T scanner (Discovery MR750 3.0 T, GE Healthcare, Milwaukee, WI, USA). The prostate MRI protocol included axial T_2_-weighted imaging and DWI with a small FOV of the prostate, axial T_1_-weighted imaging, DWI and DCE imaging of the whole pelvis in agreement with Prostate Imaging Reporting and Data System (PI-RADS) guidelines [2, 5, 6] and an additional coronal three-dimensional T_2_-weighted imaging of the lower abdomen and pelvis. Our prostate MRI protocol is described in further detail in Table 1. All patients rectally self-administered a laxative cleansing enema (Freka-Clyss^®^ 133 ml, Fresenius Kabi) 15 min prior to the exam and were given scopolamine butylbromide (Buscopan^®^, 20 mg, Sanofi-Aventis) intravenously immediately prior to the exam to mitigate image artifacts. All examinations were performed without an endorectal coil for signal reception.

**Table 1:** MRI protocol and sequence parameters

MRI protocol and sequence parameters								
*Sequences*	*Focus T*_*2*_ *-weighted imaging with restricted FOV on the prostate*	*Focus DWI imaging with restricted FOV on the prostate*	*T*_*1*_ *-weighted imaging of the whole pelvis*	*DWI imaging of the whole pelvis*	*T*_*2*_ *-weighted imaging of the lower abdomen and pelvis*	*DCE imaging of the whole pelvis*	*Post contrast T*_*1*_ *-weighted imaging of the whole pelvis*	
*Pulse sequence*	Fast spin echo	Spin echo EPI	LAVA FLEX	Spin echo EPI	Cube	DISCO	Propeller fat-saturated	
*Acquisition plane*	Axial	Axial	Axial	Axial	Coronal	Axial	Axial	
*FOV (mm* ^*2*^ *)*	200 × 200	220 × 110	408 × 408	360 × 360	370 × 370	260 × 260	340 × 340	
*Acquisition matrix*	300 × 300	136 × 68	300 × 340	140 × 160	332 × 332	260 × 212	352 × 352	
*Number of slices*	34	32	152	40	392	38	36	
*Slice thickness/gap (mm)*	3/0.3	3.5/0.0	3/1.5	5/0	1/0.5	3	5/0.5	
*Phase-encoding direction*	Anterior-posterior	Right-left	Anterior-posterior	Right-left	Superior-inferior	Anterior-posterior	Right-left	
*Flip angle*	111°	90°	12°	90°	90°	20°	90°	
*TE (ms)*	120	68.4	1.7	60.2	125.6	1.7	17.2	
*TR (ms)*	7912	4500	4.0	3664	2164	4.2	581	
*Echo train length (ms)*	28	1	1	1	160	1	6	
*b-values (s/mm* ^*2*^ *)*	N/A	50, 100, 200, 900, 1300, 2000	N/A	50, 500, 1000	N/A	N/A	N/A	
*Number of signal averages*	2	3, 3, 5, 12, 16, 17	1.4	2	1	0.7	1.5	
*Acquisition duration (s)*	252	864	121	187	296	262	167	

DCE: Dynamic contrast-enhanced; DWI: Diffusion-weighted imaging; FOV: Field of view; TE: Echo time; TR: Repetition time

### Additional findings

All MRI examinations were interpreted by a radiology resident and a senior radiologist with more than 18 years of experience in prostate MRI (H.C.T). Finalised radiology reports were reviewed, and AFs were recorded. No additional reading of the prostate MRI images was performed. Additional findings were defined as any pathology not located in the prostate or the seminal vesicles. Local extension of PCa to the adjacent structures, such as seminal vesicles, bladder, or rectal invasion was not considered as AFs as it may be visualised on the restricted FOV of an abbreviated protocol.

AFs were classified as related to or unrelated to PCa. Prostate cancer-related AFs included lymph node and bone metastases. The latter were considered related to PCa unless a biopsy showed another origin, or the MRI was classified as PIRADS 1–2.

Finally, AFs unrelated to PCa were also stratified by organ systems (genitourinary, gastrointestinal, musculoskeletal, vascular, soft tissue) and clinical significance. In agreement with previous studies [7–11] and according to local guidelines, three radiologists in consensus (F.P., P.K., and H.C.T.) divided AFs unrelated to PCa into three groups as having low, moderate or high significance.

- Group 1: low significance findings, not requiring any follow-up or treatment.
- Group 2: moderate significance, eventually requiring further diagnostic investigations, follow-up, or treatment.
- Group 3: high significance, requiring urgent further diagnostic investigations or treatment.

### Management change

Institutional medical records were reviewed to determine whether group 3 AFs were previously known or were newly diagnosed on prostate MRI. For newly diagnosed group 3 AFs, further diagnostic investigations (imaging, biopsy) or a change in treatment were considered a change in patient management. A management change was not considered in patients with AFs that were already known from a previous exam.

### Statistical analysis

The statistical analyses were performed by our statistical guarantor (H.N.) using R (R Foundation for Statistical Computing, Vienna, Austria) base functions with an alpha-threshold of 0.05 (i.e., analyses were considered significant when *p* < 0.05).

The relationship between age (in years) and the number of AFs unrelated to PCa was assessed with a one-sided Pearson’s correlation. One-sided Welch’s t-tests were computed to assess the difference between age groups (< 65 years old expected to be lower than ≥ 65 years old), and the difference between exams PI-RADS score (PI-RADS ≤ 3 expected to be lower than PI-RADS > 3), on the number of moderately and highly clinically significant AFs unrelated to PCa. Cohen’s d was used as the t-tests effect size.

For the age correlation, 65 was chosen as a cut-off because it corresponds to the conventional definition of an “elderly” [12] and because the same age was chosen in a comparative study [10]. Presence of PCa was indicated by a PI-RADS score greater than 3 which was used as a cut-off.

## Results

### Patients

**Fig. 1:**
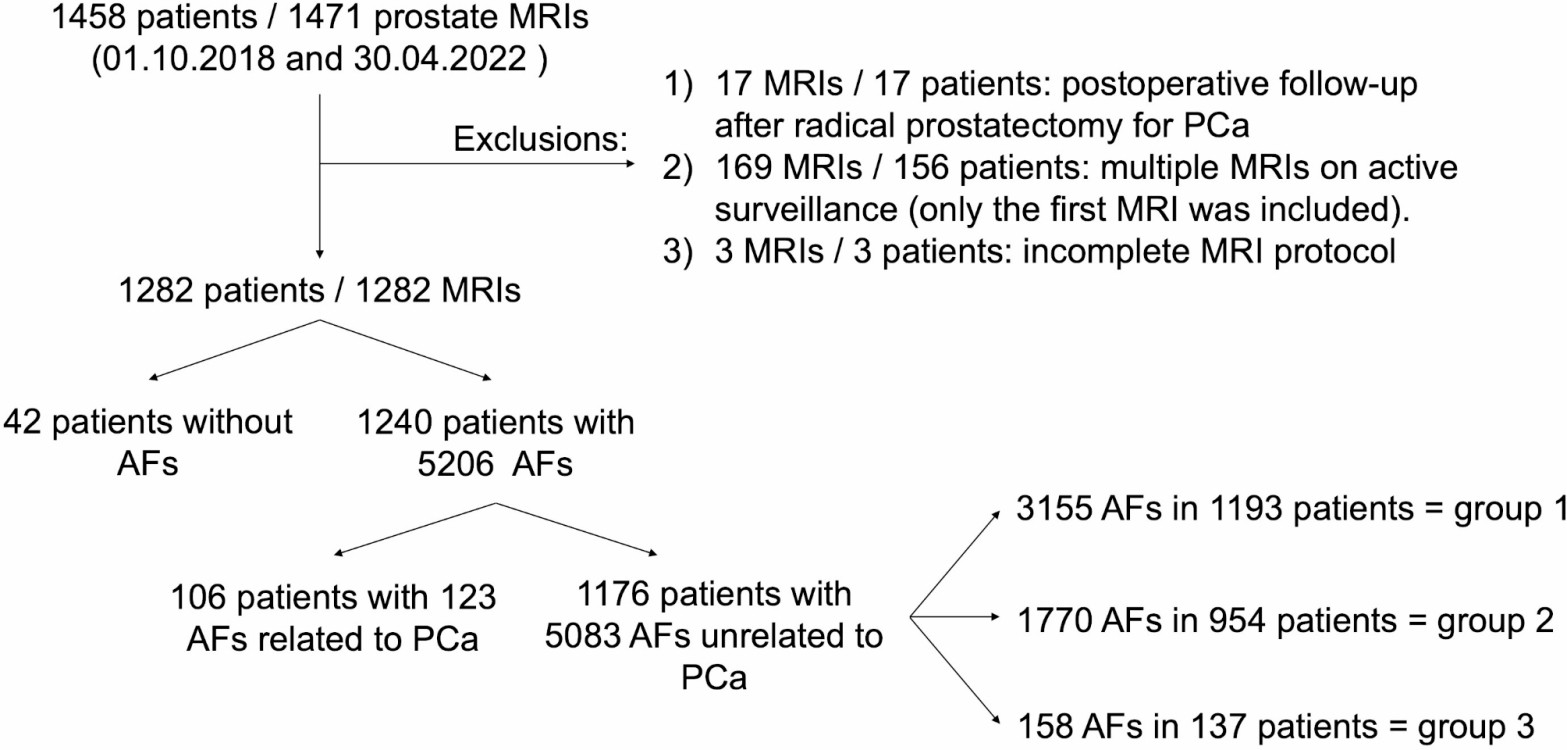
Flow diagram of the study population and distribution of additional findings (AFs) related and unrelated to prostate cancer (PCa)

A total of 1471 prostate MRI exams from 1458 patients were retrieved. Twenty MRI exams in 20 patients were excluded from analysis, 17 due to postoperative follow-up after radical prostatectomy and 3 due to an incomplete MRI protocol. One hundred and sixty-nine MRI exams in 156 patients with multiple MRIs during active surveillance were excluded, in these patients only the first MRI exam was included. Our final study population consisted of 1282 patients with the same number of MRI exams. The process of patient inclusion is shown in Fig. 1. A total number of 5206 AFs were recorded. Of 1282 patients, 42 (3.3%) patients had no AF, 90 (7.0%) patients had one AF, and 1150 (89.7%) patients had two or more AFs. The mean age was 66.8 ± 8.6 years (range, 22–94 years) with 477 (37.2%) patients aged under 65 years old and 805 (62.8%) aged 65 years or older. After reviewing finalised radiology reports, 563 (43.9%) patients had MRI exams classified as PI-RADS ≤ 3 and 719 (56.1%) PI-RADS > 3. The mean PSA level in our study population was 10.7 ± 16.6 ng/mL.

### Frequency of AFs related to PCa

A total of 123 (2.4% of all findings) extra-prostatic PCa-related AFs were found in 106 (8.3% of all patients) patients. Specifically, there was lymph node enlargement suspected for metastases in 93 (7.2%) and suspected bone metastasis in 30 (2.3%) patients.

### Frequency of AFs unrelated to PCa per organ system

**Fig. 2:**
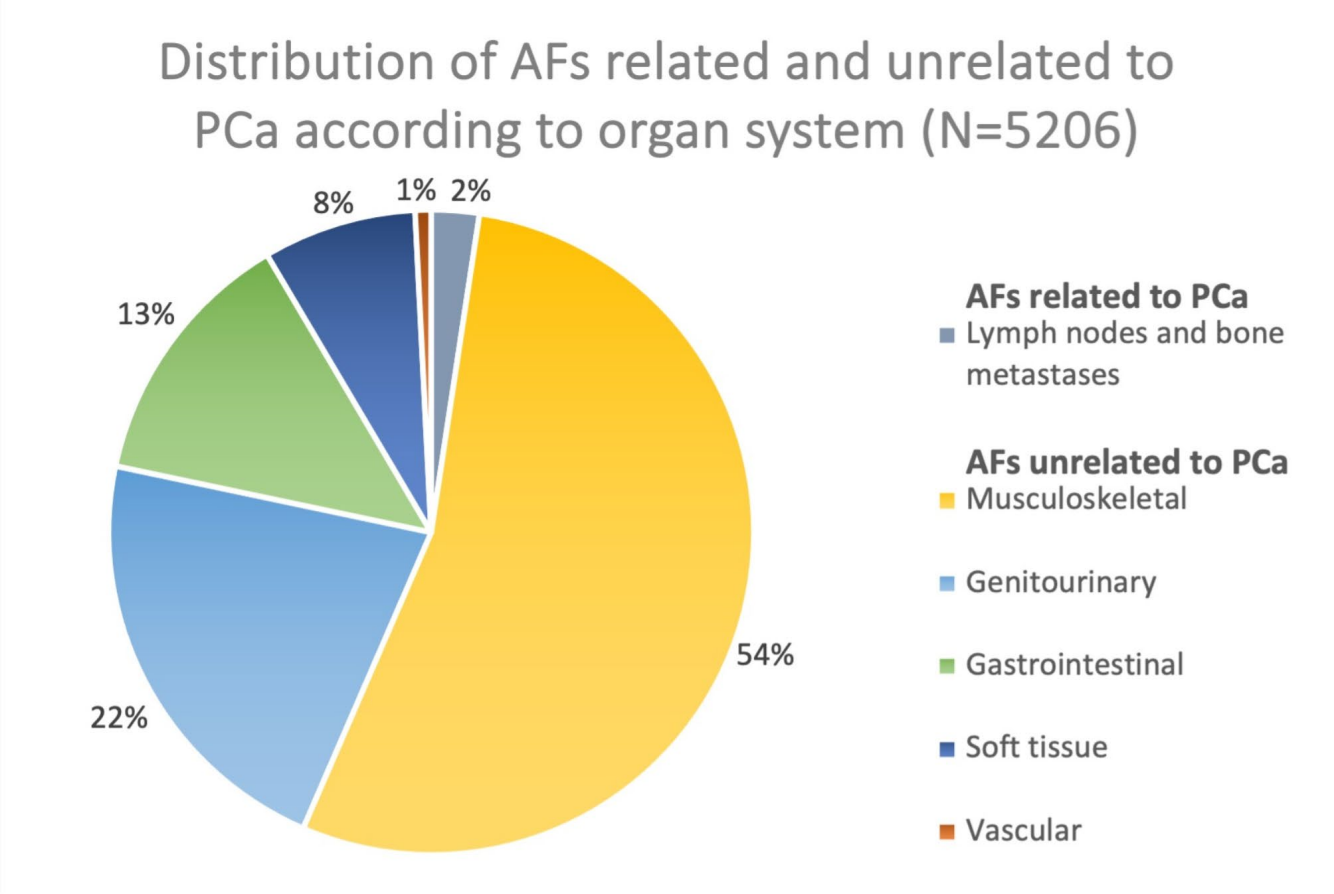
Distribution of additional findings (AFs) related and unrelated to prostate cancer (PCa) according to organ system

Of the 1176 (91.7% of all patients) patients presenting with 5083 (97.6% of all findings) AFs unrelated to PCa, the organ system with the most AFs was the musculoskeletal system (*n* = 2817, 54.1%). The two other most frequently affected systems were the genitourinary (*n* = 1135, 21.8%) and the gastrointestinal systems (*n* = 685, 13.2%). The distribution and frequency per system of AFs unrelated to PCa is reported in Fig. 2; Table 2.

**Table 2:** Prevalence of additional findings (AFs) unrelated to prostate cancer (PCa) according to organ system

Prevalence of AFs unrelated to PCa according to organ system					
*Group 1*	*N*	*Group 2*	*N*	*Group 3*	*No.*
***Genitourinary***					
Renal cysts (simple)	514	Hydrocele	60	Suspicious renal mass	44
Urachal remnant	192	Renal atrophy	14	Suspicious bladder mass or wall thickening	10
Bladder outlet obstruction	148	Urolithiasis	8	Hydronephrosis	5
Bladder diverticula	94	Varicocele	2	Adrenal gland mass	8
Genitourinary system anatomic variants	35			Pyelonephritis	1
***Gastrointestinal***					
Diverticulosis	533	Intraabdominal free liquid	56	Suspicious bowel mass or wall thickening	9
Benign hepatic lesion	67			Rectal perforation	2
Cholecystolithiasis	8			Suspicious pancreatic lesion	1
Benign splenic lesion	6				
Hemochromatosis	2				
Adenomyomatosis of gallbladder	1				
***Musculoskeletal***					
Intervertebral disc degeneration	929	Coxarthrosis	574	Fracture	14
Osteochondrosis	142	Foraminal stenosis	226	Suspicious bone lesions	8
Benign bone lesions	122	Spinal canal stenosis	181		
Tarlov cyst	119	Sacroiliac arthrosis	79		
Facet joint arthrosis	83	Bone marrow reconversion	75		
Spondylolisthesis	60	Enthesitis	49		
Scoliosis	50	Bursitis	42		
Schmorl hernia	29	Femoral head avascular necrosis	14		
Tendinopathy	10	Peripheral nerve sheath tumors	5		
Pubic arthrosis	4	Femoroacetabular impingement	1		
		Paget disease	1		
***Vascular***					
Vascular anatomic variants	7			Aneurysm	38
***Soft tissue***					
		Abdominal hernia	370	Suspect lymph node	15
		Lipoma	11	Suspicious soft tissue lesion	2
		Complicated hernia	2	Abscess	1

AFs: Additional findings; PCa: Prostate cancer

### Distribution of AFs unrelated to PCa according to clinical significance group and clinical implications

**Fig. 3:**
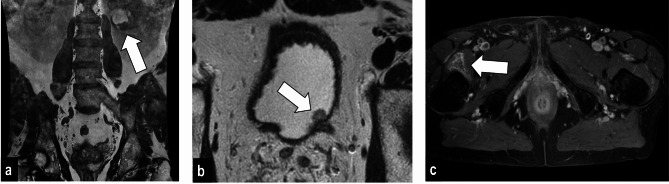
Clinically significant findings (group 3) in three different patients. **A,** 66-year-old man. Coronal T2-weighted MRI demonstrates a complicated cortical cystic lesion of the lower pole of the left kidney, showing septations and soft tissue thickening, Bosniak lV (arrow). Clear cell renal cell carcinoma was diagnosed on biopsy. **B,** 68-year-old man. Axial T2-weighted MRI shows a 8 × 7mm2 sessile bladder wall lesion (arrow); an urothelial carcinoma of the bladder has been confirmed after transurethral resection. **C,** 77-year-old man. Axial post contrast T1-weighted fat-saturated MRI shows an ill-defined, heterogeneously contrast-enhancing mass centred on the distal insertion of the iliopsoas muscle. Histology demonstrated a myxofibrosarcoma

Of the total 5083 AFs unrelated to PCa, 3155 (60.6% of all findings), 1770 (34.0%), and 158 (3.0%) were assigned to groups 1, 2, and 3, respectively. Among these, the most common finding was intervertebral disc degeneration (*n* = 929, 17.8%) belonging to group 1. The most frequent AFs in group 3 were suspicious renal lesions (*n* = 44, 0.8%), aneurysms (*n* = 38, 0.7%) and lymph node enlargement (*n* = 15, 0.3%). Examples are shown in Fig. 3.

**Fig. 4:**
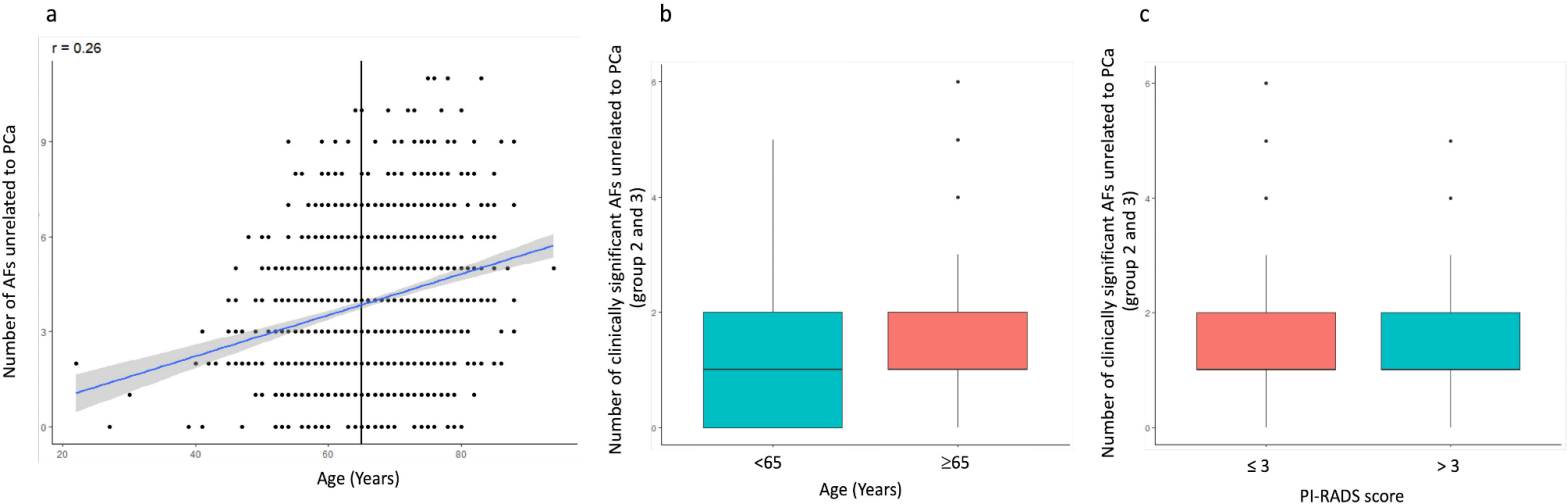
Charts of correlations between patients’ age (**a** and **b**), PI-RADS score (**c**) and number of additional findings (AFs) unrelated to prostate cancer (PCa)

The patient’s age at the time of the MRI significantly correlated with the number of AFs unrelated to PCa (*r* = 0.26, *p* < 0.001). There was a significant difference in the distribution of moderately and highly clinically significant (groups 2 and 3) AFs between patients aged under 65 years and aged 65 years or older (< 65 years old: 1.30 ± 1.15, ≥ 65 years old: 1.60 ± 1.25; Cohen’s d = -0.27; *p* < 0.001). The PI-RADS score was not significantly associated with the number of moderately and highly clinically significant AFs (PI-RADS ≤ 3: 1.49 ± 1.24, PI-RADS > 3: 1.51 ± 1.2; Cohen’s d = -0.01, *p* = 0.40). Correlation between AFs, age and PI-RADS score are illustrated in Fig. 4.

**Fig. 5:**
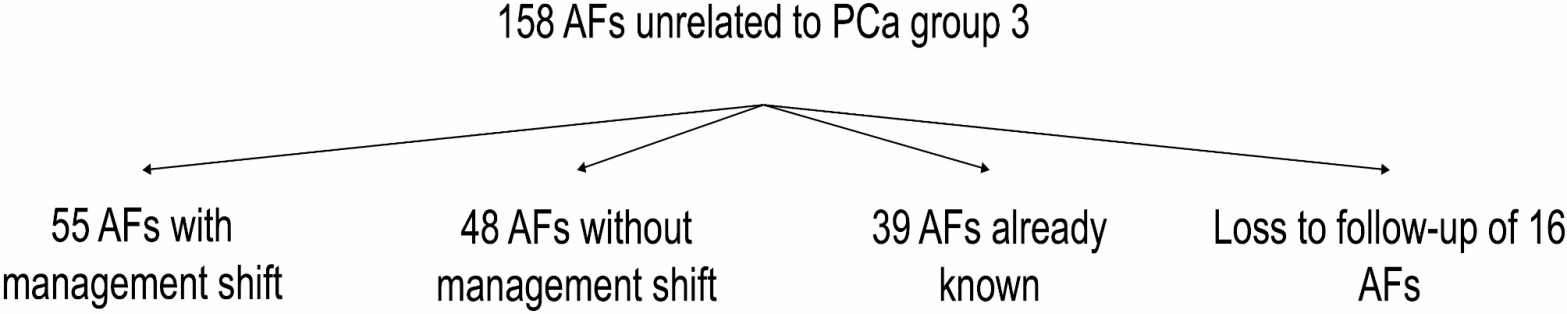
Distribution of additional findings (AFs) unrelated to prostate cancer (PCa) group 3

A change in patient management was identified in 49/137 patients of group 3 (35.8% of patients of group 3) with 55/158 (34.8% of AFs of group 3) highly significant AFs. Further diagnostic investigation included biopsy, cystoscopy, colonoscopy, PET-CT, cystography, dedicated MRI, CT, or ultrasound. Of these patients, 19 (13.9%) underwent treatment (surgical or other, see Table 3). To our knowledge, 48 AFs of group 3 (30.4%) did not result in a management change. After reviewing medical records, 39 group 3 AFs (24.7%) were already known. We lost follow-up of 16 AFs (10.1%) in group 3 (referring physician’s retirement, relocation, or death of the patient), see Fig. 5.

**Table 3:** Clinically significant findings of group 3 with additional investigations and final diagnosis and/or treatment

Clinically significant findings of group 3 with additional investigations and final diagnosis and/or treatment					
*AFs unrelated to PCa*	*N*	*Additional investigations*	*N*	*Final diagnosis and/or treatment*	*N*
***Genitourinary***					
Renal mass or cyst	25	CT	16	Renal clear cell carcinoma treated by partial nephrectomy	4
		MRI	6		
		US	2	Papillary renal cell carcinoma treated by radiofrequency ablation	1
		Biopsy	1		
				Bosniak category IIF cysts	1
				Complicated cyst	1
				Benign	18
Bladder mass or wall thickening	4	Cystoscopy	4	Urothelial carcinoma treated by transurethral resection	3
				Inflammatory pseudotumor	1
Adrenal mass	3	CT	3	Adenoma	3
Hydronephrosis	1	Cystography	1	Neurogenic bladder	1
***Gastrointestinal***					
Suspicious bowel thickening	3	Colonoscopy	3	Adenoma treated by endoscopic resection	2
				Diverticulitis treated by sigmoidectomy	1
Rectal perforation	2	CT	2	Antibiotic therapy	2
***Musculoskeletal***					
Suspicious bone lesion	1	MRI	1	Benign	1
***Vascular***					
Aneurysm	9	CT angiography Doppler ultrasound	6 3	Surgical treatment	1
				Endovascular treatment	1
***Soft tissue***					
Lymph node enlargement	6	Biopsy	3	Non-Hodgkin lymphoma treated by chemotherapy	3
		PET-CT	2		
		CT	1	HIV/AIDS lymphadenopathy treated by tritherapy	1
				Benign	2
Suspicious soft tissues lesion	1	Biopsy	1	Myxofibrosarcoma treated by radiation therapy	1

AFs: Additional findings; PCa: Prostate cancer

### MRI acquisition time

The expected acquisition time for our extended prostate MRI protocol is 36 min and 4 s. The average effective acquisition time for our population was 41 min and 36 s (standard deviation: 4 min and 29 s; range: 35 min and 13 s to 57 min and 34 s. The acquisition times depended mainly on the size of the patient and the need to repeat the acquisition of a sequence of sub-optimal quality for interpretation.

## Discussion

The results of our study show that AFs are common in male patients undergoing an extended prostate MRI protocol, with the vast majority presenting at least one AF (*n* = 1240, 96.7% of all patients). A substantial percentage (8.3%) of patients have extra-prostatic PCa-related AFs that change the patient’s disease stage and management. Many AFs unrelated to PCa are clinically significant (group 2 and 3; *n* = 1770 (34.0% of all findings), and *n* = 158 (3.0%), respectively) and may provide additional clinically relevant information. However, relatively few of these patients had AFs that led to a management shift (*n* = 49, 3.8% of all patients). Although the average number of moderately and highly clinically significant (groups 2 and 3) AFs per patient is significantly different between patients aged under 65 years and aged 65 years or older (< 65 years old: 1.30 ± 1.15, ≥ 65 years old: 1.60 ± 1.25, *p* < 0.001), the effect size is relatively small (Cohen’s d = -0.27) not justifying the use of an extended protocol only in patients 65 years or older. The number of moderately and highly clinically significant AFs was not correlated with the PI-RADS score, suggesting that patients benefit from an extended protocol regardless of the presence of PCa.

To the best of our knowledge, there are no previous studies reporting AFs found using an extended prostate MRI protocol with a coronal three-dimensional T2-weighted acquisition, which includes imaging of the abdomen that would not be seen using restricted FOV imaging as part of an abbreviated MRI protocol. Two studies have reported incidental findings related and unrelated to PCa with extended prostate MRI protocols using a two-dimensional axial T_1_-weighted sequence of the abdomen and a post-contrast abdominopelvic T_1_-weighted sequence, respectively. McEvoy et al. and Sherrer et al. [8, 9] recorded 4 (1.1%) and 119 (17.4%) incidental findings related to PCa in 355 and 684 MRIs, respectively, one with a lower percentage and the other with a higher percentage than in our study. The lower frequency of incidental findings reported by McEvoy et al. [8] may be explained by the fact that only findings identified on the T_1_-weighted sequence of the abdomen and not of the pelvis were analysed. Additionally, they only reported lymph node metastases and not bone metastases. Unlike the current study, Sherrer et al. [9] also considered local extension of PCa to adjacent structures as incidental findings, leading to a somehow artificial increase in incidental findings.

In addition to McEvoy et al. and Sherrer et al. [8, 9] who evaluated both incidental findings related and unrelated to PCa, Cutaia et al. [10] only assessed incidental findings on prostate MRI unrelated to PCa. The studies reported percentages of MRI exams with at least one incidental finding unrelated to PCa ranging from 23.1 to 52.7%, lower than in the present study [8–10]. In their studies, the percentage of incidental findings not related to PCa that were clinically significant ranged from 12.7 to 23.0%, also lower than in the present study [8–10]. We believe that our three-dimensional imaging sequence of the abdomen and pelvis allowed a more comprehensive evaluation leading to the detection of more AFs. A change in patient management was identified in 8.2% of all patients by McEvoy et al., higher than in our study [8]. Our lower rate of change in patient management may be explained by the fact that we only looked for further investigation or treatment in patients belonging to group 3, and did not consider cases where the AF was already known from a previous exam.

The acquisition time for our extended prostate MRI protocol is more than double that of an abbreviated protocol (36 min and 4 s). For comparison purposes, the abbreviated prostate protocol including only T_2_-weighted and DWI imaging on an axial plane with a restricted FOV of the prostate would last only 16 min and 36 s. Note that our protocol acquired DWI with multiple b-values and may be further shortened by reducing the number of b-values. The monetary cost of an extended prostate protocol is approximately double that of an abbreviated protocol. The difference in price is not primarily determined by the acquisition time, but mainly caused by the administration of contrast medium. It is important to keep in mind that AFs detected using an extended prostate protocol are associated with time and monetary costs. Regarding alternative protocols and imaging modalities for distant staging, a full-body MRI would extend acquisition time and would be more expensive. PSMA PET/CT is only reimbursed for very specific indications in patients with high-risk PCa. We performed the extended MRI protocol regardless of PCa risk for all our patients. In our institution, the two approaches are complementary and provide a complete assessment of distant prostatic metastases.

This study has several limitations. Firstly, the study was performed retrospectively. Secondly, the classification of AFs according to clinical significance varies from previous studies. As this classification is not standardised, it may introduce a certain bias in the comparison of the different studies. Finally, we were unable to assess the indirect benefits of AFs in terms of improved patient care and costs. However, we would like to highlight that AFs related to PCa and AFs unrelated to PCa representing coexisting comorbidities are influential factors in making treatment choices in newly diagnosed PCa [13, 14]. Furthermore, occult malignancies additionally found can be even more significant than PCa. In the current study, patients with tumours other than PCa were identified at early stages at the time of diagnosis. It is reasonable to assume that early diagnosis and in turn treatment leads to a better result and reduces follow-up costs, as treatment is less complicated in early-stage disease. This is without considering the costs of a possible additional systemic therapy and follow-up. Costs could have been further reduced if an AF explained a patient symptomatology unrelated to PCa, thus avoiding a new investigation. On the other hand, AFs can also lead to patient anxiety, iatrogenic morbidity, and increased costs. Further studies with large cohorts are needed to evaluate the cost-effectiveness of an extended versus an abbreviated prostate MRI protocol.

## Conclusion

The extended prostate MRI protocol shows a considerable prevalence of AFs of which more than a third are clinically significant, related or unrelated to PCa (groups 2 and 3). A substantial percentage (8.3%) of patients have extra-prostatic PCa-related AFs, which alter disease stage, patient prognosis and therapeutic options. Moreover, some AFs that are unrelated to PCa and belong to group 2 and 3 may explain patients’ symptoms or even alter patients’ management. However, a change in management is observed in less than 4% of all patients because of an AFs that belongs to group 3. The choice between extended and abbreviated prostate MRI protocols should involve a careful consideration of the individual needs of the patient and the institution’s capacity and expertise. Further studies are needed to assess down-stream workup of AFs and resulting benefits and costs.

## Data Availability

The datasets analyzed for the current study are not publicly available due to patient privacy. The data will be shared upon reasonable request by the corresponding author.

## Abbreviations

AFs: Additional findings
DCE: Dynamic contrast enhanced
DWI: Diffusion-weighted imaging
FOV: Field of view
MRI: Magnetic resonance imaging
PCa: Prostate cancer
PI-RADS: Prostate Imaging Reporting and Data System
PSA: Prostate specific antigen
